# Developing Novel Biointerfaces: Using Chlorhexidine Surface Attachment as a Method for Creating Anti‐Fungal Surfaces

**DOI:** 10.1002/gch2.202100138

**Published:** 2022-03-08

**Authors:** Jack A. Bryant, Lily Riordan, Rowan Watson, Naa Dei Nikoi, Wioleta Trzaska, Louise Slope, Callum Tibbatts, Morgan R. Alexander, David J. Scurr, Robin C. May, Felicity de Cogan

**Affiliations:** ^1^ Institute of Microbiology and Infection University of Birmingham Birmingham B15 2TT UK; ^2^ School of Biosciences University of Birmingham Birmingham B15 2TT UK; ^3^ Advanced Materials and Healthcare Technologies Division School of Pharmacy University of Nottingham Nottingham NG7 2RD UK

**Keywords:** antimicrobial surfaces, fungi, surface coatings

## Abstract

There is an increasing focus in healthcare environments on combatting antimicrobial resistant infections. While bacterial infections are well reported, infections caused by fungi receive less attention, yet have a broad impact on society and can be deadly. Fungi are eukaryotes with considerable shared biology with humans, therefore limited technologies exist to combat fungal infections and hospital infrastructure is rarely designed for reducing microbial load. In this study, a novel antimicrobial surface (AMS) that is modified with the broad‐spectrum biocide chlorhexidine is reported. The surfaces are shown to kill the opportunistic fungal pathogens *Candida albicans* and *Cryptococcus neoformans* very rapidly (<15 min) and are significantly more effective than current technologies available on the commercial market, such as silver and copper.

## Graphical abstract

Transmission of disease is a global problem. In the current climate, there is a significant focus on airborne transmission. However, in many infections, surfaces play a key role in transmission. The present study demonstrates the efficacy of an antimicrobial surface technology in killing opportunistic fungal pathogens.

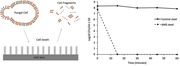

## 1 Introduction

Between 7 and 10% of patients in a healthcare setting will acquire an infection during or after their stay and the prevalence of hospital‐acquired infections (HAIs) can be as high as 51% of patients in intensive care units (ICUs).^[^ ^1^ ^]^ The recent COVID‐19 pandemic has highlighted the risk and impact of HAIs, with the rate of hospital‐acquired SARS‐CoV‐2 infection at hospitals providing acute and general care being 12–15%.^[^ ^2^, ^3^ ^]^ While the majority of non‐COVID‐19 related HAIs are caused by antimicrobial resistant bacterial pathogens, up to 20% of HAIs are caused by fungal species, many of which are able to survive for long periods on surfaces.^[^ ^4^, ^5^ ^]^ Globally, fungal diseases kill more than 1.5 million people and affect almost 1 billion with this being exacerbated by the COVID‐19 pandemic causing a rise in fungal infection cases associated with COVID‐19 patients.^[^ ^6^, ^7^, ^8^ ^]^ Among the major fungal pathogens, *Candida* species account for the majority of mucosal infections, including approximately 700 000 invasive infections globally each year.^[^ ^9^, ^10^ ^]^ Whereas, *Cryptococcus* species are the cause of cryptococcal meningitis in AIDS/HIV patients, infecting 1 million annually with mortality exceeding 50%.^[^ ^6^, ^7^ ^]^ Despite the large impact these species have, fungal diseases represent an understudied source of HAI that requires additional focus, including the development of technologies aimed at preventative measures.

Prior to the last decade, the surface environment was assumed to play a negligible role in the spread of infectious agents within the hospital environment. However, more recent evidence has demonstrated that the surface environment plays a role in the transmission of important pathogens and the spread of antimicrobial resistance.^[^ ^11^, ^12^, ^13^, ^14^ ^]^ Infectious SARS‐CoV‐2 has been shown to be stable on stainless steel surfaces, a common material of choice in the hospital environment, for >28 days at 20 °C.^[^ ^15^ ^]^ Further to this, pathogenic *Escherichia coli* is able to survive on stainless steel at room temperature for >28 days^[^ ^16^ ^]^ and *Candida albicans* for >7 days.^[^ ^17^ ^]^ While the survival of *Cryptococcus neoformans* hasn't been tested on solid surfaces, it is known to survive for >30 days on various textile fibres.^[^ ^18^ ^]^ Thus, surfaces represent a potential route through which to prevent the spread of pathogenic organisms and potentially curb the spread of antimicrobial resistance on surfaces. Besides cleaning regimens, current antimicrobial surface technologies used to combat the spread of HAIs within healthcare settings take one of three strategies to prevent or reduce microbial contamination: incorporation of a biocide to be released or mediate contact‐dependent killing, anti‐adhesive approaches or a combination of these two approaches.^[^ ^19^, ^20^, ^21^ ^]^ Antimicrobial surfaces incorporating a biocide can contain active eluting agents such as copper surfaces or materials impregnated with silver ions.^[^ ^21^ ^]^ However, both antimicrobial copper and silver ion technologies can have limited efficacy, especially when employed in the hospital environment, due to differences in surface type and the time required for killing.^[^ ^22^ ^]^ Another emerging technology includes surfaces that kill microorganisms when activated by light,^[^ ^23^ ^]^ but the requirement to be in direct sunlight limits the applicability of these systems. Due to these limitations, the uptake of these technologies into the healthcare setting has been limited.

We recently demonstrated the ability to bond an antimicrobial peptide to a steel surface, which imparted antimicrobial properties upon the treated surface when tested against both Gram‐positive and Gram‐negative bacteria.^[^ ^24^ ^]^ Stainless steel is the material of choice in the majority of healthcare settings, which led us to our decision to demonstrate the direct bonding of antimicrobials to this material in particular. While we have previously demonstrated that these antimicrobial surfaces (AMS) are active against known bacterial pathogens, the efficacy of this technology against opportunistic fungal pathogens has yet to be investigated. Here, we demonstrate the feasibility of chemically bonding an alternative broad‐spectrum antimicrobial, chlorhexidine, to steel surfaces. We then demonstrated the capacity of this material to effectively kill fungal pathogens within minutes. This work shows that the antimicrobial surface technology we have developed previously can be modified with an alternative biocide and that the antimicrobial surfaces are active against the majority of pathogenic microorganisms encountered in the healthcare environment.

## 2 Results

### 2.1 Surface Coating and Characterization

**Figure 1:**
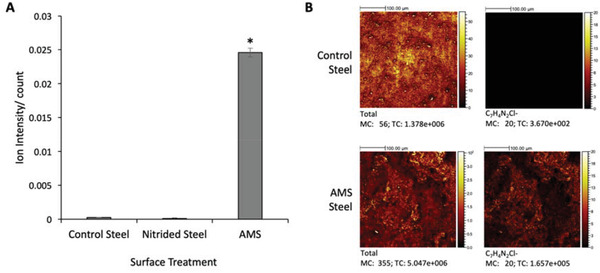
AMS analysis using ToF‐SIMS. ToF‐SIMS 4 × 4 mm scan of AMS stainless steel A). Quantification of the C_7_H_4_N_2_Cl‐ peak (*m/z*–151). Error bars show standard deviation, * denotes statistical significance of < 0.05, *n* = 3. B). ToF‐SIMS images of control or AMS steel showing a 300 μM × 300 μM region. Total ion count and the C_7_H_4_N_2_Cl‐ count are shown.

We have previously shown that nitriding can be used as a method of surface activation to allow a peptide to be bonded to an inert surface.^[^ ^24^ ^]^ Here, we have expanded this principle to create a platform technology in which nitrided surfaces can be used to incorporate organic molecules other than antimicrobial peptides. In this work, we have used chlorhexidine as a representative to demonstrate that the steel surface, following nitride activation and incubation with DIEA and HBTU catalysts, can be functionalized in a variety of ways beyond the use of antimicrobial peptides. Surfaces were nitrided, cleaned and incubated with chlorhexidine in a coating mixture modified from that reported previously to facilitate surface coating with chlorhexidine to create the AMS.^[^ ^24^ ^]^ The presence and distribution of chlorhexidine was then confirmed using time‐of‐flight secondary ion mass spectrometry (ToF‐SIMS). Analysis of the AMS showed a peak at m/z 151, which corresponds to the C_7_H_4_N_2_Cl^–^ fragment ion that is associated with chlorhexidine (**Figure** **1**A).^[^ ^25^ ^]^ This peak was only detected on surfaces functionalized with chlorhexidine, therefore confirming chlorhexidine coating and a lack of chlorhexidine on both of the control surfaces. Analysis of ToF‐SIMS chemical images showed chlorhexidine to be distributed uniformly across the whole surface of AMS steel (Figure 1B).

**Figure 2:**
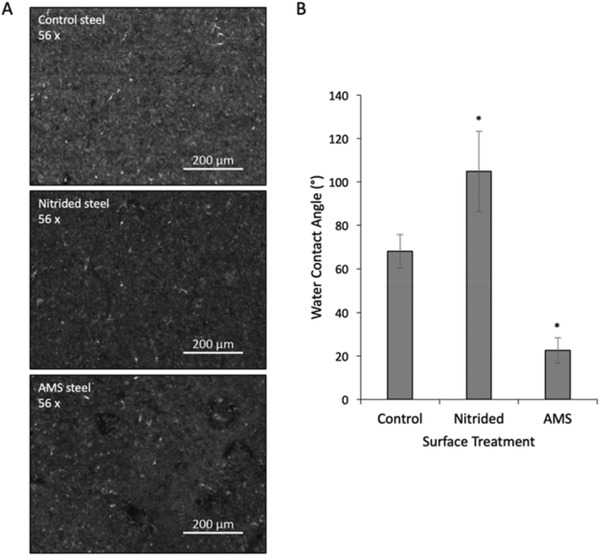
Surface properties of AMS steel. A) White light stereo microscopy of control steel, nitrided steel and AMS steel surfaces at 56 × magnification demonstrating minimal large scale surface property changes through treatment. B) Water contact angle of surfaces at different stages of the surface treatment. Error bars show standard deviation, *n* = 9 and * denotes statistical significance <0.05.

Having confirmed successful coating of the surfaces with chlorhexidine we then imaged the surfaces using low magnification light microscopy to assess any potential large scale imperfections from the coating process. Comparison of the control steel, nitrided steel and AMS steel at 56 × magnification demonstrated no major structural defects from any stage of the treatment process. Darkening of the nitrided surfaces was observed upon visual inspection and this is corroborated by microscopy images. Some surface deposits were also noted on the AMS upon visual inspection, however, these were removed by gentle washing in deionized water prior to imaging (**Figure** **2**A). We then analyzed the hydrophobicity of the surface at different stages of the treatment process to understand how the treatment affects surface properties. Water contact angle analysis showed that nitriding the surface significantly increases hydrophobicity with a water contact angle of 105 ± 19°, compared to the control untreated surface, which was 68 ± 8° (Figure 2B). However, once chlorhexidine was applied to the surface, the AMS had a water contact angle of 23 ± 6°, which demonstrates a significant reduction of hydrophobicity compared to the control untreated surfaces (Figure 2B). Therefore, full treatment of the surface leads to a decrease in hydrophobicity when compared to the untreated surface.

### 2.2 Anti‐Fungal Efficacy of AMS Steel

**Figure 3:**
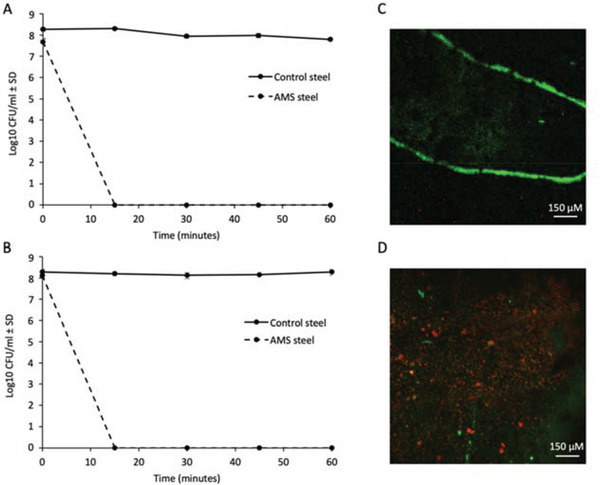
Antifungal efficacy of AMS steel. Average CFU mL^−1^ of A) *C. albicans* and B) *C. neoformans* surface inoculum over 60 min exposure to control steel or AMS steel. Fungi were cultured in 5 mL YPD broth at 30 °C for ≈18 h with shaking (180 rpm) before being adjusted to ≈1 × 10^8^ CFU mL^−1^. Cultures were pipetted onto test surfaces as 9 × 1 µl drops in a simulated splash test (≈1 × 10^6^ cells per surface) and incubated at room temperature for the designated time before cells were recovered, diluted and grown on potato dextrose agar for determination of CFU. Control steel samples are represented by a solid line, and AMS steel samples by a dashed line. Each data point represents the average of three independent assays with error bars showing standard deviation. Each experiment is representative of three independent experiments. C) Live/dead confocal microscopy of *C. albicans* incubated for 30 min on control steel or AMS steel D). Scale bars are 150 µm, and images are representative of all images collected from samples within the treatment group. Green fluorescence indicates live cells, whereas red fluorescence indicates dead cells.

Having established that chlorhexidine can be used to stably coat stainless steel, we next assessed the anti‐fungal efficacy of stainless steel AMS against the opportunistic fungal pathogens *C. albicans* and *C. neoformans*. Killing was assessed over the course of 60 min using uncoated stainless steel as a control. Cells were added to the surface at ≈1 × 10^8^ CFU (colony forming units) mL^−1^, and recovered at intervals throughout the time course (**Figure** **3**A,B).

The AMS steel demonstrated complete killing of *C. albicans* and *C. neoformans* within 15 min, whereas no significant changes in survival were observed for either organism exposed to the uncoated control steel over the time course (Figure 3A,B).

Anti‐fungal efficacy was also monitored using live/dead staining and fluorescence microscopy. Cells were stained and fixed after 30 min before being imaged on the surface by confocal fluorescence microscopy. Cells applied to control stainless steel were shown to be impermeable to the Live‐or‐Dye dead cell indicator, demonstrating survival on the control surface (Figure 3C). However, cells on the AMS surfaces accumulated the Live‐or‐Dye dead cell indicator signifying that the AMS surface had successfully killed the fungal cells applied within 30 min (Figure 3D). On neither surface was there migration outside of the area of application within the 30 min incubation window. However, the pattern of cell distribution within the dried droplet appeared to be affected by surface treatment. Cells aggregated around the droplet periphery when applied to the control surfaces, whereas a more even distribution was observed on AMS steel, likely because of changes in surface hydrophobicity.

**Figure 4:**
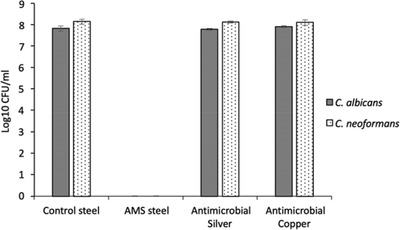
Comparison of AMS steel to commercially available antimicrobial surfaces. Antifungal effect of AMS surfaces compared to commercially available surfaces. Fungi were cultured in 5 mL YPD broth at 30 °C for ≈18 h with shaking (180 rpm) before being adjusted to ≈1 × 10^8^ CFU mL^−1^. Cultures were pipetted onto test surfaces as 9 × 1 µl drops in a simulated splash test (≈1 × 10^6^ cells per surface) and incubated at room temperature for the 30 min before cells were recovered, diluted and grown on potato dextrose agar for determination of CFU. Each data point represents the average of three independent assays with error bars showing standard deviation. Each experiment is representative of three independent experiments.

Having confirmed the anti‐fungal efficacy of the AMS stainless steel, we then compared it to commercially available technologies: copper surfaces and silver ion impregnated surfaces. To compare anti‐fungal efficacy of the different surfaces, *C. albicans* or *C. neoformans* were added to each surface at ≈1 × 10^8^ CFU mL^−1^ and CFU were quantified after 30 min incubation (**Figure** **4**). Recovered CFU for the commercial silver ion‐impregnated surface and antimicrobial copper surfaces were comparable to the untreated steel control demonstrating no measurable anti‐fungal effect of these technologies during the time course used in this experiment. In contrast, the AMS steel demonstrated complete killing of both *C. albicans* and *C. neoformans* within the 30 min incubation. This shows that in the time‐frame used for this assay the AMS steel is 100% effective in killing both *C. albicans* and *C. neoformans*, whereas the copper and silver ion impregnated surfaces were ineffective.

## 3 Conclusion

Here we demonstrated that the AMS technology we have reported previously can be modified and used with other antimicrobial active molecules such as chlorhexidine.^[^ ^24^ ^]^ This is of benefit because chlorhexidine is a broad spectrum biocide that is active against organisms from all domains of life,^[^ ^26^ ^]^ including opportunistic fungal pathogens, as demonstrated here. The AMS stainless steel was shown to have good modification across the entire surface and has been shown to be resistant to leaching of the antimicrobial from the surface,^[^ ^24^ ^]^ which is an essential requirement for AMS to resist frequent cleaning regimes and usage in the hospital environment.

The inclusion of hydrophobicity into materials has been used to alter microbial survival.^[^ ^27^ ^]^ Increasing the hydrophobicity of a surface is typically targeted at reducing initial microbial adhesion to the surface, therefore minimizing the likelihood of biofilm formation, but it can also behave to impart biocidal activity on the surface.^[^ ^20^ ^]^ However, the AMS reported here has decreased surface hydrophobicity when compared to the control steel surfaces. While the full role of surface hydrophobicity is not fully understood in this system, it is unlikely to play a strong role in the antimicrobial efficacy. In this instance, we expect that the increased surface contact area of the drops in simulated splash tests would increase the likelihood of microorganism contact with the surface bonded chlorhexidine, facilitating killing.

For AMS to be effective in the field they should rapidly kill microorganisms following contact in order to minimize cross‐contamination from high‐frequency touch surfaces, such as door handles. Chlorhexidine coated AMS demonstrated quick killing, in under 15 min, whereas commercially available antimicrobial copper, or silver ion impregnated surfaces, were ineffective within this time frame. Other studies have reported kill times of fungi on copper surfaces of 60 min,^[^ ^28^ ^]^ or 120 min,^[^ ^29^ ^]^ and up to several days for silver surfaces.^[^ ^30^ ^]^ However, the time required for effective reduction of the microbial burden does depend on the formulation of copper antimicrobial surface employed and can be as short as 30–60 s, as demonstrated for a recently developed engineered copper material that relies on increased surface area exposure through meso‐scale porosity and surface roughness.^[^ ^31^ ^]^ These longer kill times could explain why in the time interval used for this study, these surfaces are relatively ineffective by comparison to AMS stainless‐steel and this is likely related to the differences in mechanism of action. While antimicrobial copper and silver ion impregnated surfaces require release of metal ions and subsequent uptake by microorganisms to mediate killing, chlorhexidine is bonded to the AMS and not released, therefore being unlikely to kill via this mechanism.^[^ ^26^, ^32^, ^33^ ^]^ While the precise mechanism of cell killing by the AMS reported here remains to be elucidated, common mechanisms of resistance to chlorhexidine among Gram‐negative bacteria involve modifications to the outer membrane profile and the acquisition or increased expression of multidrug efflux pumps.^[^ ^34^, ^35^ ^]^ The lack of biocide release from the surface could potentially facilitate the maintenance of antimicrobial efficacy against resistant strains relying upon multidrug efflux pumps, however this would require further exploration. In conclusion, this novel AMS shows promise in the prevention of surface transmitted infections, not just from bacterial pathogens as demonstrated previously,^[^ ^1^ ^]^ but also from opportunistic fungal pathogens therefore targeting the majority of organisms causing HAIs.^[^ ^1^, ^4^, ^5^ ^]^

## 4 Experimental Section

All materials were sourced from Sigma Aldrich, UK, unless otherwise stated. Fungal strains were kindly provided by the host‐pathogen interaction research group at the University of Birmingham, UK.

### Antimicrobial Surface Formation

Steel surfaces were obtained and nitrided commercially by Rubig GmbH, Austria. Following nitriding, the surfaces were cleaned using acetone and then acetonitrile. The surfaces were then incubated in coating mixture (Acetonitrile, 52 mM 2‐(1H‐benzotriazol‐1‐yl)‐1,1,3,3‐tetramethyluronium hexafluorophosphate (HBTU) (Scientific Laboratory Supplies, Hessle, UK), 10% N,N‐diisopropylethylamine (DIEA) and 0.33% chlorhexidine digluconate (CHDG)) 6 mL cm^−2^ of surface with continuous agitation for 16–18 h. Surfaces were then removed and washed with acetonitrile followed by acetone before being air‐dried. Control samples were washed with acetonitrile and acetone then air‐dried.

### ToF‐SIMS

3D OrbiSIMS mass spectrometry imaging was performed using a HybridSIMS instrument (ION‐TOF GmbH) employing a 30 keV Bi_3_^+^ primary ion source (0.3 pA target current) in delayed extraction mode to analyze the AMS samples. Data was acquired over a 4 × 4 mm area, with a resolution of 100 pixels per mm with 15 shots per pixel. The data acquisition and analysis was performed with SurfaceLab7 (ION‐TOF GmbH). Charge neutralization was performed using a relatively low energy (<2 eV) electron floodgun.

### Water Contact Angle

Surfaces were prepared as described above. A 15 µL drop of water was applied to the surface of untreated steel and AMS steel. The drop was imaged and the contact angle was measured using Image J. Six surfaces were used per treatment and the angle was measured at both sides of each drop. The data was analyzed using SPSS (IBM).

### Stereo Microscopy

Surfaces were imaged using a Zeiss AxioZoom.V16 with top illumination using an LED ring and a Zeiss Axiocam 503 mono camera. Surfaces were briefly washed with deionized water to remove salt deposits then imaged at 56× magnification with an exposure time of 3.65 ms.

### Live/Dead Staining

For live/dead staining, the surfaces were inoculated with 1000 cells of *C. albicans* in potato dextrose broth. The surfaces were incubated at room temperature (RT) for 30 min. Surfaces were washed twice with PBS and stained using a fixable live/dead staining kit (Live‐or‐Dye, Fixable Live/Dead Staining Kit – #31064 Biotium, UK). The surfaces were fixed using 4% paraformaldehyde and then stored at 4 °C for 16 h. Surfaces were then imaged using a Leica SP8 confocal microscope using a 20×/0.7 dry objective. For imaging of live fungi, the surfaces were excited with a 488 nm laser line and images collected using a 497–558 nm emission filter. For imaging of dead stained fungi, surfaces were excited at 561 nm and images collected using a 567–701 nm emission filter. Channels were imaged sequentially and 512 × 512 pixel images were acquired at a scan speed of 400 Hz with no frame or line averaging and no accumulation.

### Antifungal Efficacy

*Candida albicans* SC5314 and *Cryptococcus neoformans* H99 were kindly provided by the Host and Pathogen Interactions group at the University of Birmingham. Fungi were cultured in 5 mL YPD broth (1% yeast extract, 2% bacto‐peptone, 2% glucose) overnight at 30 °C for ≈18 h with shaking (180 rpm) before being adjusted to ≈1 × 10^8^ CFU (colony forming units) mL^−1^. Both the AMS steel and stainless steel control were placed in a 12‐well tissue culture plate (Corning, UK). Cultures were pipetted onto test surfaces as 9 × 1 µl drops in a simulated splash test (≈1 × 10^6^ cells per surface). Following a timed incubation on the surface at room temperature, each surface was transferred into 10 ml Dey‐Engley's neutralising solution with 7–10 zirconium oxide beads and vortexed for 60 s each. Following this, the solution was serially diluted in sterile PBS and survival assessed by counting CFU after incubation at 30 °C for either ≈16 h for *C. albicans* or ≈40 h for *C. neoformans* on potato glucose agar. Both the control and AMS surface testing was repeated in triplicate for each of the fungal species.

### Comparison of AMS with Commercially Available Antimicrobial Products

Pieces of commercially available antimicrobial copper and silver ion surfaces were cut into 1 cm^2^ pieces and *C. albicans* or *C. neoformans* was applied as described for the AMS. Following a 30 min incubation on the surface at room temperature, each surface was transferred to 10 ml Dey‐Engley's neutralizing solution with 7–10 zirconium oxide beads and then vortexed for 60 s each. Following this, the solution was serially diluted in PBS and fungal survival assessed by counting CFU after incubation at 30 °C for ≈16 h for *C. albicans* or ≈40 h for *C. neoformans* on potato glucose agar.

Acknowledgement

This work was funded by a Royal Academy of Engineering Enterprise Fellowship and a Birmingham Fellowship awarded to FdC. The authors acknowledge the financial support for this work by the Engineering and Physical Sciences Research Council (EPSRC) Grant code: EP/P029868/1.

## Conflict of Interest

F.d.C. is named on a Patent which describes this invention. She is also the founder of a spin out company from the University of Birmingham based on this technology. The other authors have no conflict of interests to declare.

## Data Availability

The data that support the findings of this study are available from the corresponding author upon reasonable request.
